# Rapid Identification of Corn Sugar Syrup Adulteration in Wolfberry Honey Based on Fluorescence Spectroscopy Coupled with Chemometrics

**DOI:** 10.3390/foods12122309

**Published:** 2023-06-08

**Authors:** Shengyu Hao, Jie Yuan, Qian Wu, Xinying Liu, Jichun Cui, Hongzhuan Xuan

**Affiliations:** 1 School of Physical Science and Information Technology, Liaocheng University, Liaocheng 252059, China; haoshengyu@lcu.edu.cn; 2 School of Life Sciences, Liaocheng University, Liaocheng 252059, China; 2010150207@stu.lcu.edu.cn (J.Y.); 2110150205@stu.lcu.edu.cn (Q.W.); 3 Animal Product Quality and Safety Center of Shandong Province, Jinan 250010, China; sdfengjian@sina.com; 4 School of Chemistry and Chemical Engineering, Liaocheng University, Liaocheng 252059, China; cuijichun@lcu.edu.cn

**Keywords:** wolfberry honey, adulteration, fluorescence spectroscopy, fluorescence lifetime, principal component analysis

## Abstract

Honey adulteration has become a prominent issue in the honey market. Herein, we used the fluorescence spectroscopy combined with chemometrics to explore a simple, fast, and non-destructive method to detect wolfberry honey adulteration. The main parameters such as the maximum fluorescence intensity, peak positions, and fluorescence lifetime were analyzed and depicted with a principal component analysis (PCA). We demonstrated that the peak position of the wolfberry honey was relatively fixed at 342 nm compared with those of the multifloral honey. The fluorescence intensity decreased and the peak position redshifted with an increase in the syrup concentration (10–100%). The three-dimensional (3D) spectra and fluorescence lifetime fitting plots could obviously distinguish the honey from syrups. It was difficult to distinguish the wolfberry honey from another monofloral honey, acacia honey, using fluorescence spectra, but it could easily be distinguished when the fluorescence data were combined with a PCA. In all, fluorescence spectroscopy coupled with a PCA could easily distinguish wolfberry honey adulteration with syrups or other monofloral honeys. The method was simple, fast, and non-destructive, with a significant potential for the detection of honey adulteration.

## 1. Introduction

Honey has been a functional food for centuries due to its nutrients and therapeutic effects [1]. Honey is rich in various chemical components, including monosaccharides (fructose and glucose) and other types of sugars; these are the dominant constituents, accounting for 70–80% of honey [2]. It also contains small amounts of amino acids (including tryptophan, tyrosine, and phenylalanine), proteins (enzymes), vitamins (especially vitamin B6, thiamine, niacin, riboflavin, and pantothenic acid), minerals (including calcium, copper, iron, magnesium, manganese, phosphorus, potassium, sodium, and zinc), phenolic acids (caffeic acid, ferulic acid, chlorogenic acid, and vanillin acid), flavonoids (galangin, chrysin, apigenin, pinobanksin, naringenin, and quercetin) and royal jelly aliphatic acids [3,4,5]. It is because honey contains these minor materials that it differs from syrups and other sweeteners; thus, these substances are also the basis for the authentication identification of honey adulteration.

Honey adulteration with various syrups such as corn syrup, sugarcane syrup, beet syrup, rice syrup, wheat syrup, inverted syrup, and inulin syrup has become a prominent issue that harms the interests of consumers and beekeepers [2,3,6]. With the increase in the types of adulterated honey, other technologies are needed to detect adulterated honey [2]. The traditional methods mainly include pollen identification, which identifies the sources of the nectar and plants through the characteristics and quantity of the honey pollen, and characteristic parameters such as the water content, Brix, electrical conductivity, amylase value, and 5-hydroxymethylfurfural content [7,8,9]. However, these methods are often time-consuming and labor-intensive, and have advanced requirements for technicians. Modern analytical techniques, including ultra-performance liquid chromatography-quadrupole time-of-flight mass spectrometry (UPLC-QToF MS) [10], gas chromatography (GC-MS) [11], a stable carbon isotopic ratio analysis (SCIRA) [12], high-performance anion exchange chromatography (HPAEC) [13], nuclear magnetic resonance (NMR) [14], and e-noses and e-tongues have also been employed for the detection of syrup in adulterated honey [15,16], but these methods or techniques are either complicated to operate, or the detection process and sample pre-processing are time-consuming or expensive. For example, SCIRA can be used for the C4 plant sugar corn syrup adulteration in honey, but the instrument is expensive and complicated to operate.

Fluorescence spectroscopy is a type of spectral detection technology that has rapidly developed in recent years because of its simple operation as well as it being fast and non-destructive [17,18]. The theory basis for fluorescence spectroscopy is that when a fluorescent molecule absorbs photons, it changes from an original ground state to an excited state. The excited-state molecule consumes part of its energy by colliding with the surrounding molecules and rapidly drops to the lowest vibration level of the first electron excited state, remaining there for about 10^−9^–10^−7^ s. After that, the excess energy is directly released in the form of photon and drops to various vibration levels of the electronic ground state. At this time, the emitted light is fluorescent and can be detected using fluorescence spectrometer [19].

Honey is rich in vitamins, phenols, polypeptides, and amino acids (tryptophan, tyrosine, and phenylalanine) as well as other fluorophores, thus front-face fluorescence spectroscopy and three-dimensional (3D) synchronous fluorescence spectroscopy have been used in honey authentication of botanical origin and geographical origin [20,21,22,23,24,25]. However, few studies have been reported on the authenticity of honey by combining multiple fluorescence spectroscopy techniques, especially the authenticity of honey by measuring fluorescence lifetime. When a substance is excited by a laser beam, the molecules absorb energy and leap from the ground state to a certain excited state, and then fluoresce back to the ground state in the form of a radiative leap. When the excitation light is removed, the time required for the fluorescence intensity of the molecule to drop to 1/e of the maximum intensity of the fluorescence at the time of excitation is called the fluorescence lifetime.

Differences in the fluorophores between monofloral honeys of different botanical origin and geographical origin or between monofloral and multifloral honeys indicate different fluorescent characteristics. Similarly, the fluorescent characteristics of adulterated honey must change compared with authentic honey. Thus, it is possible to detect monofloral honey, multifloral honey, or adulterated honey by fluorescence spectroscopy.

Wolfberry honey is a typical monofloral honey that is mainly produced in Northwest China, including Ningxia, Qinghai, and Gansu provinces [14]. The nectar plant of wolfberry honey is wolfberry, a traditional health food with a variety of pharmacological activities. Customers have shown an increasing interest in wolfberry honey due to its potential health benefits [26]. Due to its high price, natural wolfberry honey, just like other monofloral honey, is easy to be adulterated in the honey market, and the common adulteration techniques involve sugar syrup adulteration, or adding multifloral honey to monofloral honey, or nectar adulteration. Thus, the rapid identification of wolfberry honey adulteration is an urgent need. Here, the authenticity of wolfberry honey was determined by combining multiple fluorescence spectroscopy techniques mainly from the maximum fluorescence intensity, peak positions, and fluorescence lifetime, and these data were further analyzed by a principal component analysis (PCA), which demonstrated that fluorescence spectroscopy was a simple, fast, and non-destructive method for the detection of honey adulteration.

## 2. Materials and Methods

### 2.1. Materials

A total of 23 wolfberry honey samples were collected from different apiaries in 2021 of Golmud in Qinghai Province, Northwest of China. Multifloral honey samples were obtained from different beekeepers in the counties of Meiyuan, Gangcha, and Datong of Qinghai Province.

An acacia honey, only as a monofloral honey control sample, was collected from an apiary in the Shandong Province of North China in 2021. Corn syrup was obtained from the Daesang Corporation of Korea, and corn maltose syrup (M50) was from the Hubei Hefeng Grain and Oil Group Co., Ltd., Wuhan, China. All samples were stored at 4 °C before use.

### 2.2. The Characteristic Parameters Analysis of Wolfberry Honey Samples

The water content, total sugar content, and Baume degree were determined using a honey refractometer (Shanghai Lichen Instrument Technology Co., Ltd., Shanghai, China). The pH values were measured by using a pH meter (Shanghai Yidian Instrument Technology Co., Ltd., Shanghai, China). The total protein content was tested using the standard Bradford’s method. The diastase (α-amylase) activity was measured according to GB/T 18932 (China). The conductivity of dissolved honey solution was determined using an electrical conductivity instrument (Shanghai Leici Instrument Technology Co., Ltd., Shanghai, China).

### 2.3. Palynological Identification

Pollen grains in the wolfberry honey samples were obtained according to our previous publication [27]. In brief, sugar was first removed from the honey samples by centrifugation twice at 12,000 g for 15 min. The sediment was then resuspended with 1 mL glutaraldehyde solution (2.5%) overnight. After that, all samples were gradient dehydrated with ethanol and freeze-dried in a vacuum. The palynological identification was taken using a Hitachi S-750 SEM system (Hitachi Company, Tokyo, Japan).

### 2.4. Preparation of the Test Samples

All samples were diluted with distilled water to contain 30 mg per milliliter and were exhausted with an ultrasonic cleaner (40 kHz; 80 W; 30 s) prior to analysis.

Adulterated samples were prepared by adding 10%, 20%, 30%, 40%, 50%, 60%, 70%, 80%, and 90% acacia honey, corn syrup, and corn maltose syrup to the wolfberry honey.

### 2.5. Fluorescence Spectroscopy Detection

#### 2.5.1. Fluorescence Spectra Measurement

The fluorescence spectra measurements of the different samples were determined with an Edinburgh F900 with a R928-P detector (Edinburgh Instruments, EI, Livingston, UK), and a 1 cm quartz cell was used in the measurement. The fluorescence intensity of each sample solution was directly measured with an excitation wavelength of 280 nm and an emission wavelength range from 300 to 540 nm with a 1 nm increment, and spectrometer slits were set for a 2.5 nm band-pass. The fluorescence intensity of all samples were recorded at room temperature.

#### 2.5.2. Three-Dimensional Fluorescence Spectra Measurement

The 3D fluorescence spectra of the different samples were measured with an F-7000 FL spectrophotometer (Hitachi, Japan), and a 1 cm quartz cell was used in the measurement. The instrument parameters were an excitation wavelength from 200 to 450 nm and an emission wavelength from 260 to 560 nm; scan speed was 30,000 nm/min; excitation slit and emission slit were all set for 2.5 nm; PMT voltage was 600 V. The 3D fluorescence spectra of all samples were recorded at room temperature.

#### 2.5.3. Fluorescence Lifetime Measurement

The fluorescence lifetime was tested using an Edinburgh F900 at an excitation wavelength of 280 nm and an emission wavelength of 350 nm. The light source was 900 µF. The average fluorescence lifetime was calculated according to our previous publication [19].

### 2.6. Statistical Analysis

The statistical analysis involved a paired Student’s *t*-test, Tukey test, and an ANOVA using SPSS version 18.0. A *p*-value less than 0.05 was considered to indicate a statistically significant difference. Origin Pro8 software was also used for the fluorescence spectra data processing, mapping analysis, and principal component analysis.

## 3. Results and Discussion

### 3.1. Characteristic Parameters of Wolfberry Honey Samples

The characteristic parameters of wolfberry honey samples can be seen in Table 1, which preliminary indicated the quality of wolfberry honey used in the present study. The pH of the wolfberry honey samples was 4.17, which was within the typical blossom honey pH range of 3.5 to 4.5. The contents of water and total sugar as well as Baume degree were consistent with the standards of the European Regulations of quality. The protein content was about 27.62 mg per 100 g, which mainly derived from plant pollen or honey honeybee itself and contributed to the main fluorophores in honey. The conductivity was about 0.34 mS/cm, less than 0.8 mS/cm specified by relevant standards. Diastase activity is an important indicator to evaluate the quality of honey, and it is related to the freshness and processing and storage conditions. The diastase activity of wolfberry honey samples was 26.18 ± 1.91 mL/(g·h), obviously higher than the European quality standards, indicating that all the wolfberry honey samples were fresh and unprocessed.

### 3.2. Palynology Characteristics of Wolfberry Honey Samples

**Figure 1:**
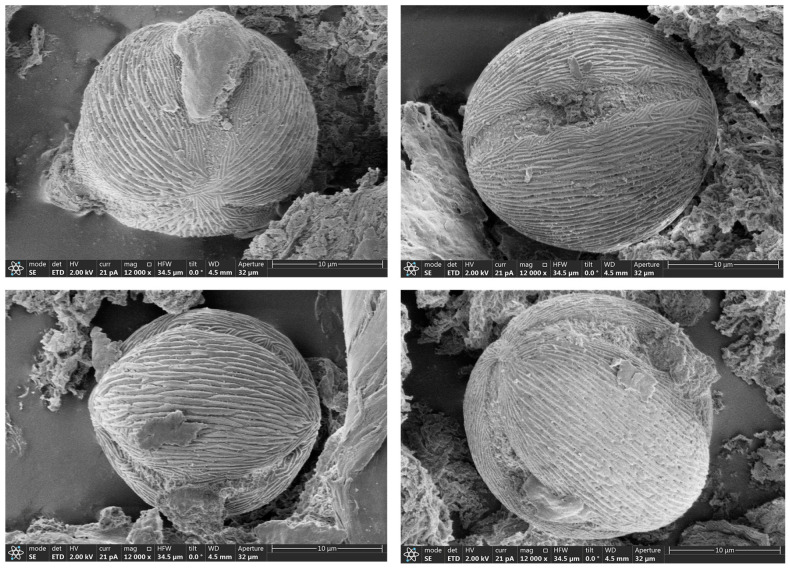
Scanning electron micrographs of pollen from wolfberry honey.

In order to determine the authenticity of the botanic source of wolfberry honey, we observed the palynology characteristics of wolfberry honey samples using scanning electron microscopy. The morphology of the pollen grains from wolfberry honey samples showed typical palynological characteristics of *Lycium Linn*., namely the morphology of pollen was subspherical, triple-grooved with grooves up to both poles, which can be found in Figure 1.

### 3.3. Fluorescence Spectra of Wolfberry Honey, Multifloral Honey and Syrups

The main components of honey are sugar and water, and it also contains small amounts of protein, free amino acids, phenolic acids, flavonoids, and minerals [4]. These minority components in honey showed fluorescent properties [22,23]. A peak with an excitation of 280 nm and emission of 340 nm can potentially suggest fluorescence from aromatic amino acids or protein in honey [28].

**Figure 2:**
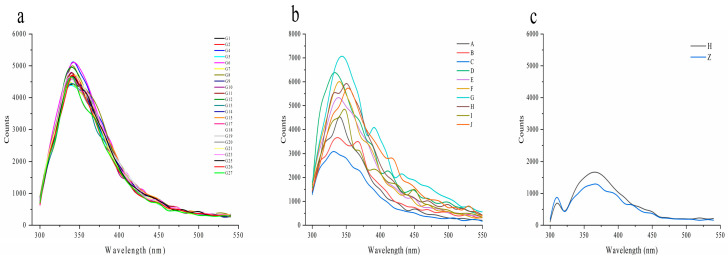
The fluorescence spectra of wolfberry honey, multifloral honey, and syrups. (**a**) The fluorescence spectra of all 23 wolfberry honey samples (G1–G27, 23 wolfberry honey samples). (**b**) The fluorescence spectra of the multifloral honey samples (A–J, 10 multifloral honey samples). (**c**) The fluorescence spectra of corn syrup and corn maltose syrup (H, corn syrup, Z, corn maltose syrup).

The fluorescence spectra of wolfberry honey, multifloral honey, and syrups at a fixed excitation wavelength of 280 nm can be found in Figure 2. The fluorescence spectra of all 23 wolfberry honey samples were consistent with similar fluorescence intensity and peak positions, indicating the typical fluorescence spectra of the wolfberry honey from the same botanical origin and geographical origin (Figure 2a), and the data were consistent with our previous study on acacia honey authenticity identification, and further confirm the accuracy of the methodology [19]. Conversely, the multifloral honey samples collected from different geographical origins including Meiyuan, Gangcha, and Datong of Qinghai province showed different fluorescence intensities and peak positions compared with those of wolfberry honey samples (Figure 2b). The main components of syrup are sugar with few fluorescent compounds such as proteins, amino acids, and phenolic acids, thus the fluorescence intensities of the two kinds of corn syrups were much lower than those of the authentic honey samples and the peak positions showed an obvious redshift (Figure 2c).

Monofloral honey, multifloral honey, and syrups showed different fluorescence spectra at a fixed excitation wavelength of 280 nm, indicating fluorescence spectroscopy is can differ the botanical origin and geographical origin of honey, and can easily distinguish honey from syrups by comparing the peak positions and fluorescence intensity.

Table 2 shows the differences in the maximum fluorescence intensity and the peak positions of the wolfberry honey samples, multifloral honey, corn syrups, and corn maltose syrup. The maximum fluorescence intensity of the wolfberry honey was 4838.49 ± 181.21, and that of multifloral honey was 4412.6 ± 305.49, both of which were significantly higher than that of the syrups. The peak position of the wolfberry honey samples was 342.65 ± 3.11 nm. However, the peak positions of the two types of syrups were completely different from those of the authentic wolfberry honey; these were 365 ± 1.41 nm and 363 ± 8.49 nm, respectively.

The difference in chemical components or fluorophores among the wolfberry honey, multifloral honey, and two kinds of corn syrups lead to different fluorescence spectra at a fixed excitation wavelength of 280 nm, which can be used to distinguish the monofloral honey, multifloral honey and syrups.

### 3.4. Fluorescence Spectra Changes in the Adulterated Wolfberry Honey

**Figure 3:**
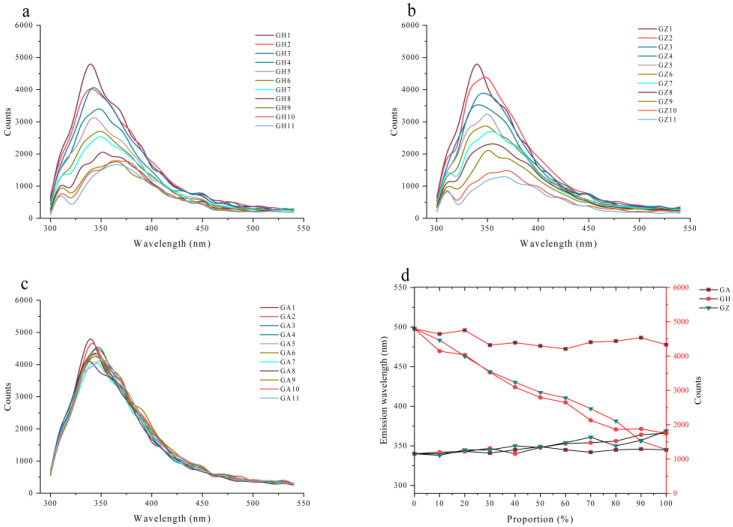
The fluorescence spectra of the adulterated wolfberry honey. (**a**) The fluorescence spectra of wolfberry honey adulterated with different proportions of corn syrup (0–100%). (**b**) The fluorescence spectra of wolfberry honey adulterated with different proportions of corn maltose syrup (0–100%). (**c**) The fluorescence spectra of wolfberry honey adulterated with different proportions of acacia honey (0–100%). (**d**) The change trends in emission wavelengths and fluorescence intensities of wolfberry honey adulterated with different proportions of syrups and acacia honey (0–100%). GA: wolfberry honey adulterated with different proportions of acacia honey; GH: wolfberry honey adulterated with different proportions of corn syrup; GZ: wolfberry honey adulterated with different proportions of corn maltose syrup.

When different proportions of corn syrup or corn maltose syrup (10–100%) were adulterated into wolfberry honey, the fluorescence intensity decreased with an increase in the syrup concentration, and the peak positions of the adulterated honey samples also redshifted (Figure 3a,b). However, if wolfberry honey was mixed with another monofloral honey, acacia honey, the characteristic properties of acacia honey can be found in Supplementary Table S1, the changes between the maximum fluorescence intensities and the peak positions were slight and not obvious compared with the genuine wolfberry honey (Figure 3c). Figure 3d showed the change trends in the peak positions and the fluorescence intensities of wolfberry honey adulterated with different proportions of syrups and acacia honey (0–100%). These results further demonstrated syrup adulteration in monofloral honey can be easily detected at a fixed excitation wavelength of 280 nm, and the maximum fluorescence intensities and the peak positions were two important parameters, but it was not easy to distinguish the mixture of two kinds of monofloral honey samples only by fluorescence spectra.

### 3.5. The 3D Fluorescence Spectra of the Wolfberry Honey Samples and Syrups

The 3D fluorescence spectroscopy is a matrix spectrum characterized by excitation wavelength (*Y*-axis), emission wavelength (*X*-axis), and fluorescence intensity (*Z*-axis), which is able to obtain the information of the excitation wavelength and emission wavelength as well as the information of fluorescence intensity. Therefore, 3D fluorescence spectroscopy is also called total luminescence spectra.

**Figure 4:**
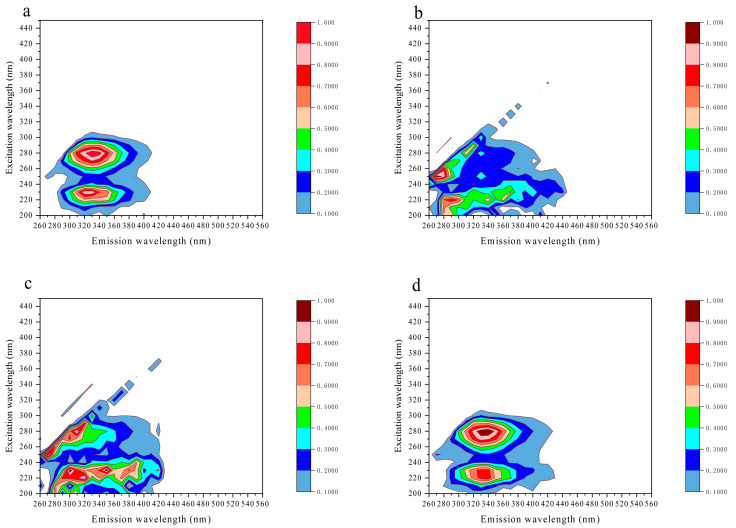
The three-dimensional (3D) fluorescence spectra of the wolfberry honey and syrups. (**a**) The 3D fluorescence spectra of wolfberry honey. (**b**) The 3D fluorescence spectra of corn syrup. (**c**) The 3D fluorescence spectra of corn maltose syrup. (**d**) The 3D fluorescence spectra of acacia honey.

The 3D fluorescence spectra of the wolfberry honey, corn syrup, and corn maltose syrup at an excitation wavelength from 200 to 450 nm and an emission wavelength from 260 to 560 nm were obviously different and could easily be distinguished (Figure 4a–c). From the 3D contour map, we can obviously see that there were two peak values at the excitation wavelength of 280 nm and 230 nm and the corresponding emission wavelength at about 340 nm, which indicated the fluorophores might be aromatic amino acids and non-flavonoid phenolic compounds in the honey sample [28,29]. The 3D fluorescence spectra of two different corn syrups were totally different compared with the wolfberry honey sample without significant peak values and peak positions, which is consistent with the results from 280 nm excitation wavelength, and the 3D contour maps can visually distinguish honey from syrups.

Similarly, it was difficult to distinguish the wolfberry honey from the acacia honey by the 3D fluorescence spectra (Figure 4d), for two kinds of monofloral honey had similar 3D fluorescence spectra with similar two peak values and peak positions. The peak positions and the maximum fluorescence intensities of the wolfberry honey, corn syrup, corn maltose syrup, and acacia honey can be found in Supplementary Table S2.

### 3.6. The 3D Fluorescence Spectra Changes in the Adulterated Wolfberry Honey

**Figure 5:**
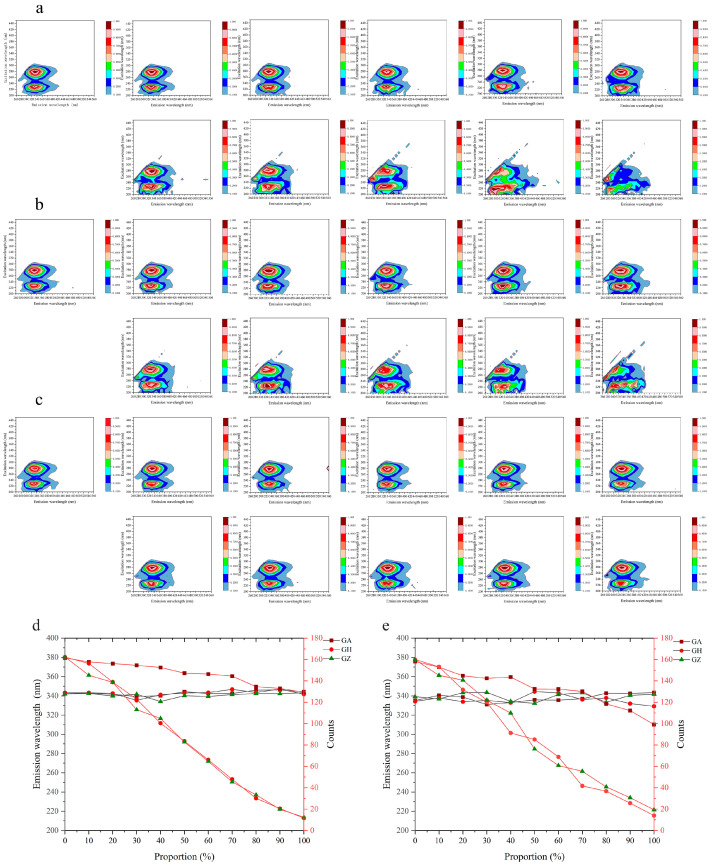
The three-dimensional (3D) fluorescence spectra of the wolfberry honey adulterated with different proportions of syrup or acacia honey. (**a**–**c**) The 3D fluorescence spectra of wolfberry honey adulterated with different proportions of corn syrup (0–100%), corn maltose syrup (0–100%) and acacia honey (0–100%). (**d**) The change trends in emission wavelengths and fluorescence intensities of wolfberry honey adulterated with different proportions of syrups and acacia honey (0–100%) at the maximum peak value. (**e**) The change trends in emission wavelengths and fluorescence intensities of wolfberry honey adulterated with different proportions of syrups and acacia honey (0–100%) at the second peak value. GA: wolfberry honey adulterated with different proportions of acacia honey; GH: wolfberry honey adulterated with different proportions of corn syrup; GZ: wolfberry honey adulterated with different proportions of corn maltose syrup.

The 3D fluorescence spectra demonstrated obvious changes in peak values and peak positions with an increase in the syrup concentration (10–100%) at an excitation wavelength from 200 to 450 nm and an emission wavelength from 260 to 560 nm, especially at excitation wavelength of 280 nm and 230 nm. However, the differences between the wolfberry honey and adulterated wolfberry honey with a different concentration of acacia honey (10–100%) were not obvious from the 3D fluorescence spectra. (Figure 5a–c), the change trends in peak position and peak value can be found in the Figure 5d,e.

### 3.7. The Fluorescence Lifetime of Wolfberry Honey and Syrups

The fluorescence lifetime is an intrinsic parameter of fluorescent substances, which is not easily disturbed by the concentration of fluorescent molecules, stray light, fluorescence scattering angle, and can visually reflect the decay process of fluorescence signal of fluorescent substances [30].

**Figure 6:**
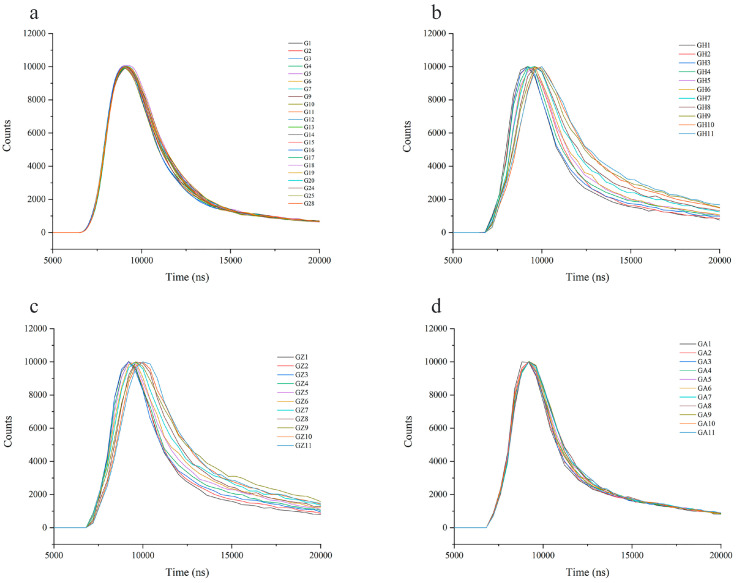
The fluorescence lifetime fitting plots of wolfberry honey and syrups. (**a**) The fluorescence lifetime fitting plots of wolfberry honey. (**b**) The fluorescence lifetime fitting plots of wolfberry honey adulterated with different proportions of corn syrup (0–100%). (**c**) The fluorescence lifetime fitting plots of wolfberry honey adulterated with different proportions of corn maltose syrup (0–100%). (**d**) The fluorescence lifetime fitting plots of wolfberry honey adulterated with different proportions of acacia honey (0–100%).

The fluorescence lifetime of the wolfberry honey and the syrups can be determined from the decay of their fluorescence intensity as a function of time. The fluorescence lifetime fitting plots of the wolfberry honey samples almost overlapped together and had no significant difference (Figure 6a), indicating that authentic wolfberry honey samples had similar fluorescence lifetime decay plot. However, the fluorescence lifetime fitting plots of the adulterated wolfberry honey samples especially in the decay domain shifted obviously compared with the authentic honey samples with an increase in the syrup concentration (10–100%) (Figure 6b,c), which indicated that the difference in the fluorescence lifetime between wolfberry honey and adulterated honey. Similar to the results of fluorescence spectra at a fixed excitation wavelength of 280 nm and 3D fluorescence spectra, the fluorescence lifetime fitting plots of honey samples adulterated with acacia honey were slightly shifted with the increase in acacia honey incorporation ratio (10–100%) (Figure 6d). The mean fluorescence lifetime of the wolfberry honey, acacia honey, and syrups can be found in Supplementary Table S3.

### 3.8. The PCA of Wolfberry Honey and Syrups

PCA is a dimensionality-reduction statistical method that is often used to reduce the dimensionality of large data sets. Seven main parameters, including the maximum fluorescence intensity and peak positions at the fixed excitation wavelength of 280 nm, two peak values, and two peak positions from the 3D fluorescence spectra, and fluorescence lifetime were used to perform the PCA. Two principal components, PC1 and PC2, were extracted according to the initial eigenvalues. The cumulative variance contribution rate reached 92.36%, which fully showed the difference between the wolfberry honey and the adulterated honey samples. In PC1, the maximum fluorescence intensity at 280 nm excitation wavelength and the peak values at 3D excitation wavelengths (200–450 nm excitation wavelength) had a positive effect, whereas the peak positions at 280 nm excitation wavelength and fluorescence lifetime had a negative effect. The fluorescence lifetime had higher loading factors in PC2, which had a positive effect on PC2. Although it was difficult to distinguish wolfberry honey from acacia honey using fluorescence spectra, they could be easily discriminated combined with PCA.

**Figure 7:**
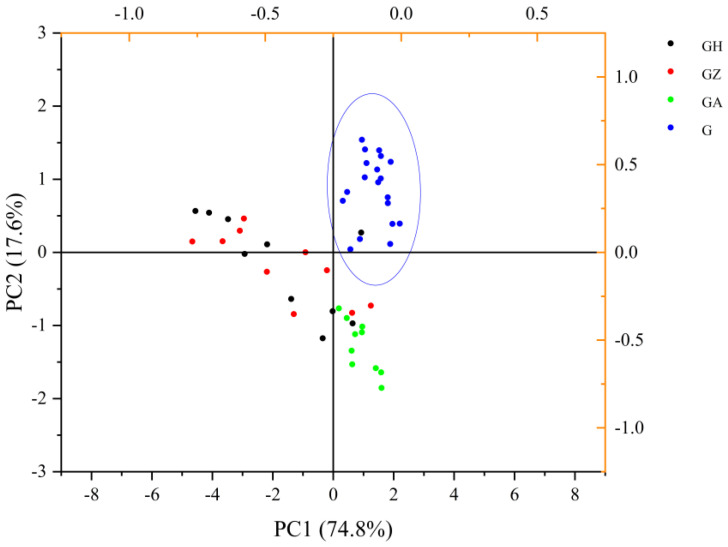
Principle components analysis of wolfberry honey and wolfberry honey adulterated with different proportions of syrups. GH: wolfberry honey adulterated with different proportions of corn syrup (10−100%); GZ: wolfberry honey adulterated with different proportions of corn maltose syrup (10−100%); GA: wolfberry honey adulterated with different proportions of acacia honey (10−100%); G: wolfberry honey samples.

A PCA Biplot was used to depict the wolfberry samples and the adulterated honey samples. The dispersions were obvious and allowed us to easily distinguish the wolfberry honey from the adulterated honey at a 10% incorporation (Figure 7). From the PCA score plot we found the determination factor to distinguish acacia honey from wolfberry honey was mainly fluorescence lifetime. The maximum fluorescence intensity and peak positions at 280 nm excitation wavelength, the peak values at 3D excitation wavelengths, and fluorescence lifetime together determined the proportion of syrups in wolfberry honey. With an increase in the syrup concentration in wolfberry, the effects of peak positions at 280 nm excitation wavelength and fluorescence lifetime were more significant.

## 4. Conclusions

We reported multiple fluorescence spectroscopy techniques combined with chemometrics can easily detect wolfberry honey adulteration with corn syrup, corn maltase syrup, and other pure monofloral honey such as acacia honey. The fluorescence intensities and peak positions are the easy-to-obtain characteristics to distinguish genuine honey from syrups because of their differences in the fluorophore concentration. The 3D fluorescence spectroscopy visually and comprehensively identified honey and syrup by obtaining the information of the excitation wavelength, emission wavelength, as well as fluorescence intensity. The fluorescence lifetime, independent of the fluorophore concentration, also can differentiate the adulterated honey samples. These results further confirmed the differences in their chemical constituents especially fluorophores of the monofloral honey, multifloral honey, syrups, and adulterated honey. Compared with other more expensive, sophisticated, time-consuming, and labor-intensive methods, the fluorescence spectroscopy detection time for each sample was only within several minutes and the pretreatment of the sample was simple. In all, fluorescence spectroscopy combined with chemometrics is a simple, fast, low-cost, and non-destructive method to quickly identify honey adulteration with corn syrup and corn maltose syrup.

## Figures and Tables

**Table 1:** Characteristic parameters of wolfberry honey.

Variables	Units	Wolfberry Honey
pH	-	4.17 ± 0.08
Water	g per 100 g	20.48 ± 0.59
Total sugar	g per 100 g	77.74 ± 0.72
Baume degree	°Bé	41.42 ± 0.25
Protein content	mg per 100 g	27.62 ± 3.01
Conductivity	mS/cm	0.34 ± 0.02
Diastase activity	mL/(g·h)	26.18 ± 1.91

**Table 2:** The comparison of honey and syrups between the maximum fluorescence intensity and peak position.

	Wolfberry Honey	Multifloral Honey	Corn Syrup	Corn Maltose Syrup
Maximum fluorescence intensity	4838.49 ± 181.21 ^a^	4412.6 ± 305.49 ^b^	1813.25 ± 94.02 ^b,c^	1400.9 ± 173.23 ^b,c^
Peak position (nm)	342.65 ± 3.11 ^b,c^	346.5 ± 2.99 ^b^	365 ± 1.41 ^a^	363 ± 8.49 ^a^

Different letters (a, b, c) in each column indicate significant differences calculated using Tukey test.

## Data Availability

The data used to support the findings of this study can be made available by the corresponding author upon request.

## Supplementary Materials

The following supporting information can be downloaded at: https://www.mdpi.com/article/10.3390/foods12122309/s1.
