# Supplementary material for: Rapid Identification of Corn Sugar Syrup Adulteration in Wolfberry Honey Based on Fluorescence Spectroscopy Coupled with Chemometrics
Source: Foods. 2023 Jun 8;12(12):2309. doi: 10.3390/foods12122309 (PMC10296839; doi:10.3390/foods12122309)
Supplement: Supplementary file 1 [file foods-12-02309-s001.zip › Supplementary Table S1.pdf]

Supplementary Table S1 Characteristic parameters of acacia honey

Variables	Units	Acacia honey
pH	-	3.79±0.01
Water	g per 100 g	17.13±0.23
Total sugar	g per 100 g	81.56±0.37
Baume degree	°Bé	43.06±0.11
Protein content	mg per100g	30.8±0.05
Conductivity	μS/cm	136.50±0.71
Diastase activity	mL/(g·h)	28.30±1.82
