# Supplementary material for: Rapid Identification of Corn Sugar Syrup Adulteration in Wolfberry Honey Based on Fluorescence Spectroscopy Coupled with Chemometrics
Source: Foods. 2023 Jun 8;12(12):2309. doi: 10.3390/foods12122309 (PMC10296839; doi:10.3390/foods12122309)
Supplement: Supplementary file 1 [file foods-12-02309-s001.zip › Supplementary Table S2.pdf]

Supplementary Table S2 The comparison of honeys and syrups between the maximum fluorescence intensities and peak positions at the excitation wavelength of 230 nm and 280 nm

	wolfberry honey		corn syrup		corn maltose syrup		acacia honey	
	230 nm	280 nm	230 nm	280 nm	230 nm	280 nm	230 nm	280 nm
Maximum fluorescence intensity	172.34± 16.78	196.03± 20.84	25.13± 7.78	43.64± 6.51	21.81± 5.30	16.27± 6.61	108.67± 13.48	131.95± 3.75
Peak position (nm)	330.00± 0.00	330.36± 1.89	300.00± 0.00	290.00± 0.00	355.00± 21.21	325.00± 7.07	335.00± 7.071	330.00± 0.00
