# Supplementary material for: Rapid Identification of Corn Sugar Syrup Adulteration in Wolfberry Honey Based on Fluorescence Spectroscopy Coupled with Chemometrics
Source: Foods. 2023 Jun 8;12(12):2309. doi: 10.3390/foods12122309 (PMC10296839; doi:10.3390/foods12122309)
Supplement: Supplementary file 1 [file foods-12-02309-s001.zip › Supplementary Table S3.pdf]

Supplementary Table S3 The mean fluorescence lifetime of different samples

	wolfberry honey	acacia honey	corn syrup	corn maltose syrup
Fluorescence lifetime	7155.45±160.52	6551.22±109.91	7360.33±18.61	7267.67±5.48
